# Inactive USP14 and inactive UCHL5 cause accumulation of distinct ubiquitinated proteins in mammalian cells

**DOI:** 10.1371/journal.pone.0225145

**Published:** 2019-11-08

**Authors:** Jayashree Chadchankar, Victoria Korboukh, Leslie C. Conway, Heike J. Wobst, Chandler A. Walker, Peter Doig, Steve J. Jacobsen, Nicholas J. Brandon, Stephen J. Moss, Qi Wang

**Affiliations:** 1 AstraZeneca Tufts Laboratory for Basic and Translational Neuroscience, Tufts University, Boston, MA, United States of America; 2 Discovery Sciences, BioPharmaceuticals R&D, AstraZeneca, Waltham, MA, United States of America; 3 Neuroscience, BioPharmaceuticals R&D, AstraZeneca, Waltham, MA, United States of America; 4 Department of Neuroscience, Tufts University School of Medicine, Boston, MA, United States of America; 5 Department of Neuroscience, Physiology and Pharmacology, University College, London, United Kingdom; Lund University, SWEDEN

## Abstract

USP14 is a cysteine protease deubiquitinase associated with the proteasome and plays important catalytic and allosteric roles in proteasomal degradation. USP14 inhibition has been considered a therapeutic strategy for accelerating degradation of aggregation-prone proteins in neurodegenerative diseases and for inhibiting proteasome function to induce apoptotic cell death in cancers. Here we studied the effects of USP14 inhibition in mammalian cells using small molecule inhibitors and an inactive USP14 mutant C114A. Neither the inhibitors nor USP14 C114A showed consistent or significant effects on the level of TDP-43, tau or α-synuclein in HEK293T cells. However, USP14 C114A led to a robust accumulation of ubiquitinated proteins, which were isolated by ubiquitin immunoprecipitation and identified by mass spectrometry. Among these proteins we confirmed that ubiquitinated β-catenin accumulated in the cells expressing USP14 C114A with immunoblotting and immunoprecipitation experiments. The proteasome binding domain of USP14 C114A is required for its effect on ubiquitinated proteins. UCHL5 is the other cysteine protease deubiquitinase associated with the proteasome. Interestingly, the inactive mutant of UCHL5 C88A also caused an accumulation of ubiquitinated proteins in HEK293T cells but did not affect β-catenin, demonstrating USP14 but not UCHL5 has a specific effect on β-catenin. We used ubiquitin immunoprecipitation and mass spectrometry to identify the accumulated ubiquitinated proteins in UCHL5 C88A expressing cells which are mostly distinct from those identified in USP14 C114A expressing cells. Among the identified proteins are well established proteasome substrates and proteasome subunits. Besides β-catenin, we also verified with immunoblotting that UCHL5 C88A inhibits its own deubiquitination and USP14 C114A inhibits deubiquitination of two proteasomal subunits PSMC1 and PSMD4. Together our data suggest that USP14 and UCHL5 can deubiquitinate distinct substrates at the proteasome and regulate the ubiquitination of the proteasome itself which is tightly linked to its function.

## Introduction

The ubiquitin-proteasome system (UPS) is the main protein degradation pathway in eukaryotic cells [1]. It determines the half-life of most cellular proteins, eliminates misfolded and damaged proteins, and is essential for protein homeostasis in cells. Proteins destined for degradation are tagged by the conjugation of a small 76-residue protein called ubiquitin, often in the form of polymeric chains, which enable the substrate to be recognized and degraded by the proteasome [2–4]. The 26S proteasome is composed of a 20S core particle (CP) containing the proteolytic chamber, and one or two 19S regulatory particles (RP) critical for substrate recognition, deubiquitination, unfolding, and translocation [5, 6]. Prior to and during substrate degradation, its ubiquitin tag must be removed, ensuring efficient substrate translocation into the proteolytic chamber and ubiquitin recycling in the cell [7, 8]. The 19S RP harbors three deubiquitinating enzymes (DUBs) including two cysteine proteases, ubiquitin-specific protease 14 (USP14 in mammals or Ubp6 in yeast) and ubiquitin C-terminal hydrolase L5 (UCHL5, also known as UCH37), and a Zn^2+^-dependent metalloprotease RPN11 [9–12].

Deubiquitination by RPN11 is tightly coupled to substrate unfolding and subsequent degeneration. Loss of RPN11 activity causes accumulation of ubiquitinated proteins and cell death in budding yeast and mammalian cells [7, 10, 13, 14]. No homolog of UCHL5 is present in the genome of budding yeast [11]. Loss of UCHL5 does not affect cell survival or change the level of free or conjugated ubiquitin [14]. Unlike RPN11 and USP14 which appear to only function at the proteasome, UCHL5 also functions in the chromatin-remodeling complex INO80 [15, 16]. Knockout of UCHL5 in mice causes prenatal lethality [17]. USP14/Ubp6 is dynamically associated with the 19S RP through its ubiquitin-like (UBL) domain, not essential for cell survival, but important for ubiquitin recycling in budding yeast and a spontaneous ataxia mouse strain [11, 18, 19]. Loss of USP14 in mammalian cells does not alter free and conjugated ubiquitin levels but increases the degradation rate [14, 20].

It was reported that USP14-mediated deubiquitination may lead to release of the otherwise competent substrate from the proteasome and prevent its degradation, therefore inhibition of USP14 may accelerate degradation of such substrates [21–24]. But this effect was not observed in other studies [25–28]. A small molecule USP14 inhibitor IU1 and an inactive USP14 mutant C114A were shown to reduce the level of the amyotrophic lateral sclerosis (ALS) and dementia disease proteins TDP-43 and tau in mammalian cells [22]. Another small molecule inhibitor of USP14 and UCHL5, b-AP15, was shown to induce an accumulation of ubiquitinated proteins and apoptotic cell death in multiple myeloma cells [29]. Therefore, IU1 data suggests that inhibiting USP14 increases the function of the proteasome while b-AP15 data suggests that inhibiting USP14 impaires the function of the proteasome.

We were interested in USP14 as a potential drug target for neurodegenerative diseases. We started our study by repeating the experiments where USP14 WT or the inactive mutant C114A was co-expressed with TDP-43, tau or α-synuclein in HEK293T cells [22, 27]. Neither USP14 WT nor C114A changed the protein level of TDP-43, tau or α-synuclein which is consistent with the data reported by Miller and colleagues [27]. Surprisingly, we observed a robust accumulation of high molecular weight K48-linked polyubiquitinated proteins with USP14 C114A expression. We reasoned these proteins are potential USP14 substrates, therefore combined immunoprecipitation and mass spectrometry analysis and identified a list of accumulated proteins including known proteasome substrates and multiple proteasome subunits. Among the identified proteins we focused on β-catenin and validated ubiquitinated β-catenin indeed accumulated with USP14 C114A expression. These data prompted us to examine the effects of UCHL5 C88A in cells. Interestingly, UCHL5 C88A expression also caused an accumulation of high molecular weight K48-linked polyubiquitinated proteins but did not affect β-catenin. Among the proteins identified by mass spectrometry, we also verified that UCHL5 C88A increased its own ubiquitination and USP14 C114A increased ubiquitination of proteasomal subunits PSMC1 and PSMD4. Both USP14 C114A and UCHL5 C88A have been commonly used in many studies, but our study for the first time clearly show the consequences of their expression in mammalian cells on ubiquitinated proteins. Our study also supports that USP14 and UCHL5 can deubiquitinate specific substrates at the proteasome and are key factors of proteasome autoregulation by deubiquitination of proteasome subunits.

## Materials and methods

### Plasmids and biochemical reagents

Plasmids containing myc-TDP-43 and myc-TDP-43 M337V were generated as described previously [30]. Plasmids for tau (4R/2N), α-synuclein, USP14 and UCHL5 were purchased from Origene. Mutant plasmids USP14 C114A, USP14 WT ΔUBL, USP14 C114A ΔUBL and UCHL5 C88A were generated from the wildtype plasmid by Genscript. Cells were treated with IU1 (25–100 μM; Selleckchem or Sigma), MG132 (10 μM; Selleckchem), PS341 (10 μM; Selleckchem) and b-AP15 (0.5–2 μM; Selleckchem or Millipore). All compounds were dissolved in DMSO (Sigma), and control samples were treated with the same volume of DMSO.

### *In vitro* USP14 ubiquitin-rhodamine hydrolysis assay

Ubiquitin-Rhodamine 110 (Ub-Rho) and ubiquitin vinyl sulfone (Ub-Vs) treated 26S-proteasomes (26S-VS) were purchased from Ubiquigent (60-0117-050 and 65-1020-BUL, respectively). Full length USP14 was purified as described previously [31]. IU1 (Sigma) and b-AP15 (Millipore) were dissolved in DMSO and dispensed into black 384 well Greiner plates (Greiner Bio, 784076) over a gradient resulting in 100 μM to 3 nM final concentrations in the assay. 5 μL of 2X USP14/26S-VS solution was added to the assay plate in an assay buffer of 40 mM Tris-HCl pH 7.5, 0.01% Brij-35, 1 mM TCEP. Reactions were started by adding 5 μL of 2X solution of Ub-Rho (2X concentration of 200 nM). Plates were covered and incubated at room temperature for 60 minutes, and the reactions were stopped by adding 5 μL of 100 mM citric acid. Plates were incubated for 15 minutes at room temperature and then read on a Tecan M1000 microplate reader (Fluorescence Intensity, Excitation wavelength: 485 nm, Excitation bandwidth: 10 nm, Emission wavelength: 520 nm, Emission bandwidth: 10 nm). IC_50_ values were determined by fitting curves to a log (compound concentration) vs. response—variable slope equation using GraphPad Prism 7.0.

### Cell culture and transfections

HEK293T cells (ATCC) were cultured in DMEM (Thermo Fisher) containing 10% FBS and 1% penicillin/streptomycin at 37°C with 5% CO2 in a humidified incubator. For transfections, cells were plated on 6-well plates or 10 cm plates 24 h prior to transfection. Cells were transfected with plasmids using Lipofectamine 2000 (Thermo Fisher) according to manufacturer’s instructions. Medium was changed 24 hours after transfection and cells were lysed 48 hours after transfection unless stated otherwise.

### Cell lysis and immunoblotting

Cells were scraped in an appropriate volume of RIPA buffer (R0278, Sigma) supplemented with a protease inhibitor cocktail (Complete Mini EDTA free tablets, Sigma-Aldrich). The cell lysate was centrifuged at 10,000 g for 10 minutes at 4°C. The supernatant was collected, and protein concentrations were measured using the BCA assay (Pierce). 20–50 μg of total proteins were mixed with LDS buffer (Thermo Fisher) containing 20% β-mercaptoethanol and heated at 55°C for 10 minutes. The samples were centrifuged briefly and separated using 4–12% Bis-Tris gels or 4–12% Bolt gels (Thermo Fisher) and transferred onto polyvinylidene difluoride (PVDF) membranes (Millipore). The blots were blocked with 5% milk or 5% BSA in 1x TBST and probed with the following antibodies: α-synuclein (10842-1-AP, Proteintech, 1:1000), α-tubulin (7291, mouse, Abcam, 1:5000), α-tubulin (52866, rabbit, Abcam, 1:5000), α-tubulin (11224-1-AP, rabbit, Proteintech, 1:3000), β-catenin (610153, BD Transduction Laboratories, 1:2000), β-tubulin (T8328, Sigma, 1:5000), FK2 (PW8810, Enzo Life Sciences, 1:10000), GAPDH (sc-32233, Santa Cruz Biotechnology Inc., 1:10000), HA tag (3724, Cell Signaling Technology, 1:2000), PSMA4 (11943-2-AP, rabbit, Proteintech, 1:500), PSMC1 (11196-1-AP, rabbit, Proteintech, 1:500), PSMC3 (13923S, rabbit, Cell Signaling Technology, 1:500), PSMD1 (PA1-973, rabbit, Invitrogen, 1:500), PSMD4 (3336S, rabbit, Cell Signaling Technology, 1:500), tau (Monoclonal MN1000, Thermo Fisher, 1:1000), TDP-43 (10782-2-AP, Proteintech, 1:5000), TfR (Transferrin Receptor/CD71 Antibody #13–6800, Thermo Fisher, 1:1000), ubiquitin (3933, Cell Signaling Technology, 1:1000), K48-linked polyubiquitin (#05–1307, Millipore, 1:1000), K63-linked polyubiquitin (#05–1308, Millipore, 1:1000), UCHL5 (sc-271002, Santa Cruz Biotechnology Inc., 1:1000), USP14 (WH0009097M4, Sigma Aldrich, 1:1000). Blots were then incubated with mouse or rabbit secondary antibodies conjugated with HRP (Jackson Immunoresearch). ECL or fluorescein-labeled secondary antibodies were used to detect immunoblotting signals. With ECL, bands were detected using ECL substrate (Super Signal West Dura, Thermo Scientific) and imaged using the BioRad Universal Hood III Imager and BioRad Image Lab software. Bands were quantified using Quantity One (BioRad) and normalized to housekeeping genes in the same samples and then all samples were normalized to the average of control samples in the same experiments. With fluorescein-labeled secondary antibodies (IRDye secondary antibodies, LI-COR), bands were imaged at 680 and 800 nm simultaneously using LI-COR Odyssey CLx imager. Bands were quantified by ImageJ. Normalization was done similarly as using ECL. We used GAPDH, α-tubulin, β-tubulin and TfR as housekeeping genes which did not change with experimental conditions.

### Immunoprecipitation

One hundred μg of proteins were mixed with RIPA buffer to a final volume of 500 μl. Protein A/G agarose beads (Pierce) were added to the above protein samples and rotated at 4°C for 1 h for preclearing. The samples were then centrifuged at 8,000 g for 1 minute. 10% of the supernatant was used as input to confirm expression and loading. 2–10 μg of specific antibodies or control mouse or rabbit IgG antibodies (Jackson Immunoresearch) were added to the remaining supernatant and rotated at 4°C for 1 h. Then fresh protein A/G agarose beads (Thermo Fisher) were added to the above samples and rotated at 4°C overnight to pulldown antibody bound proteins. The samples were centrifuged at 8,000 g for 1 minute. The beads were washed with RIPA buffer four times and the immunoprecipitated complexes were detached from the beads by boiling the samples at 95°C for 10 minutes in 30 μl of LDS loading buffer containing 5% β-mercaptoethanol. The samples were centrifuged at 10,000 g for 15 minutes and the supernatants were analyzed using immunoblotting.

### IP for mass spectrometry analysis

Scaled-up IP was performed as described above with 3700 μg of proteins. The K48 immunoprecipitates from USP14 WT, USP14 C114A, UCHL5 WT and UCHL5 C88A samples, and the control rabbit IgG immunoprecipitates from USP14 C114A and UCHL5 C88A samples were loaded on a 4–12% Bis-Tris gel (Thermo Fisher). The gel was stained using Coomassie blue (EZBlue Stain, Sigma) and destained using dH_2_O. The gel pieces above the heavy chain were cut and analyzed using mass spectrometry.

### Mass spectrometry

Trypsin digestion, liquid chromatography-tandem mass spectrometry (LC-MS/MS), and MS/MS spectral search in the human database (Uniprot) using the Sequest 28 analysis program was performed by Taplin Mass Spectrometry Facility (Harvard University).

To identify proteins specifically accumulated in USP14 C114A and UCHL5 C88A samples, proteins detected in the IgG control sample were removed without further consideration. The remaining proteins that were enriched using the Sum Intensity parameter in the C114A and C88A samples compared to the WT samples in all three replicates were included in Table 1, Table 2, S1 and S3 Tables. As control, the inverted analyses were done to identify proteins that were reduced using the Sum Intensity parameter in the C114A and C88A samples compared to the WT samples in all three replicates. These proteins were included in S2 and S4 Tables.

**Table 1:** Ubiquitinated proteins accumulated in the cells expressing USP14 C114A.

Reference number	Gene symbol	Gene name
Q9UKV8	AGO2	Argonaute-2
P49368	CCT3	T-complex protein 1 subunit gamma
P35222	CTNNB1	β-catenin
P60842	EIF4A1	Eukaryotic initiation factor 4A-I
P02751	FN1	Fibronectin
Q5JWF2	GNAS	Guanine nucleotide-binding protein G(s) subunit alpha isoforms XLas
O95373	IPO7	Importin-7
O95751	LDOC1	Leucine zipper, down-regulated in cancer 1
Q9NTJ4	MAN2C1	α-mannosidase 2C1
Q15084	PDIA6	Protein disulfide-isomerase
Q15149	PLEC	Plectin-1
P25789	PSMA4	Proteasome subunit alpha type-4
P62191	PSMC1	26S protease regulatory subunit 4
P17980	PSMC3	26S protease regulatory subunit 6A
P55036	PSMD4	26S proteasome non-ATPase regulatory subunit 4
Q86YD1	PTOV1	Prostate tumor-overexpressed gene 1
P51571	SSR4	Translocon-associated protein subunit delta
P07996	THBS1	Thrombospondin-1
P29144	TPP2	Tripeptidyl-peptidase 2
M0R2S1	UBA52	Ubiquitin-60S ribosomal protein L40
Q05086	UBE3A	Ubiquitin-protein ligase E3A
Q93008	USP9X	Probable ubiquitin carboxyl-terminal hydrolase FAF-X

**Table 2:** Ubiquitinated proteins accumulated in the cells expressing UCHL5 C88A.

Reference number	Gene symbol	Gene name
Q8IUX7	AEBP1	Adipocyte enhancer-binding protein 1
P08243	ASNS	Asparagine synthetase
Q9UBB4	ATXN10	Ataxin-10
P27797	CALR	Calreticulin
P49368	CCT3	T-complex protein 1 subunit gamma
Q66GS9	CEP135	Centrosomal protein of 135 kDa
P53618	COPB1	Coatomer subunit beta
Q13618	CUL3	Cullin 3
Q8IXB1	DNAJC10	DnaJ homolog subfamily C member 10
P02751	FN1	Fibronectin
J3QL06	HYOU1	Hypoxia up-regulated protein 1
O00410	IPO5	Importin-5
Q96P70	IPO9	Importin-9
Q7Z304	MAMDC2	MAM domain-containing protein 2
Q13765	NACA	Nascent polypeptide-associated complex subunit alpha
Q9Y2A7	NCKAP1	Nck-associated protein 1
P14543	NID1	Nidogen-1
Q15084	PDIA6	Protein disulfide-isomerase A6
Q02809	PLOD1	Procollagen-lysine,2-oxoglutarate 5-dioxygenase 1
P25789	PSMA4	Proteasome subunit alpha type-4
P17980	PSMC3	26S protease regulatory subunit 6A
Q99460	PSMD1	26S proteasome non-ATPase regulatory subunit 1
Q15019	SEPT2	Septin-2
P07996	THBS1	Thrombospondin-1
P52888	THOP1	Thimet oligopeptidase
Q9Y5K5	UCHL5	Ubiquitin carboxyl-terminal hydrolase isozyme L5
Q9NYU2	UGGT1	UDP-glucose:glycoprotein glucosyltransferase 1
P26640	VARS	Valine-tRNA ligase

### Statistics

GraphPad Prism 7.0 was used for statistical analysis. One-way analysis of variance (ANOVA) was used to test for statistical significance in all figures except Fig 7 where t-test was used to determine statistical significance. Values are mean ± standard deviation (SD). Statistical significance was set at *P* < 0.05.

## Results

### Inactive USP14 C114A does not change the level of TDP-43, tau or α-synuclein in HEK293T cells

**Fig 1:**
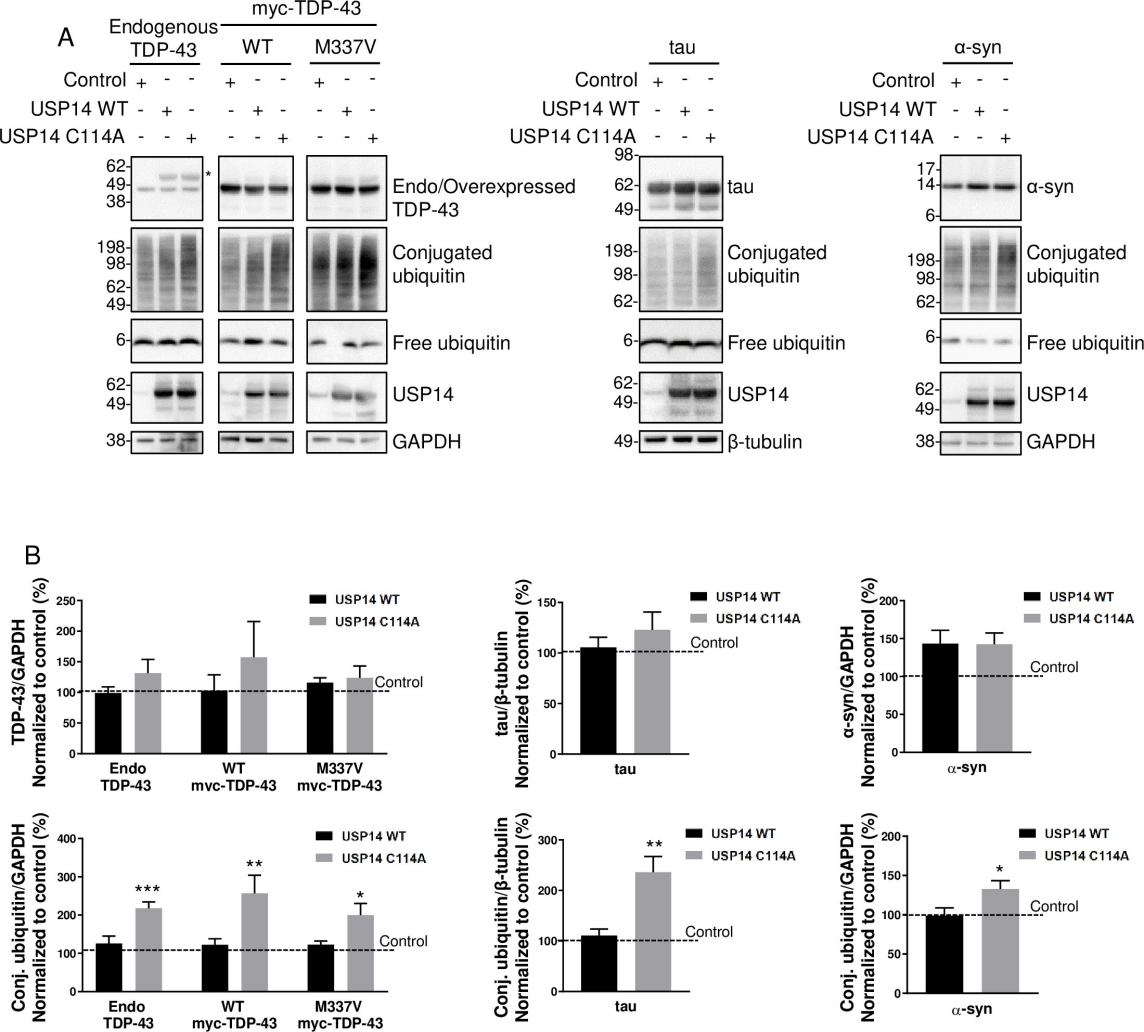
Expression of USP14 C114A has no effect on TDP-43, tau or α-synuclein but leads to an accumulation of ubiquitinated proteins. (A) Immunoblot showing the effects of a control plasmid, USP14 WT or C114A co-expression with myc-TDP-43 WT, myc-TDP-43 M337V, tau or α-synuclein in HEK293T cells. * Non-specific band. (B) USP14 WT or C114A has no effect on the protein level of TDP-43, tau and α-synuclein. Quantification of protein levels from A. n = 3–6, error bars represent SEM. USP14 C114A causes an accumulation of polyubiquitinated proteins. Quantification of conjugated ubiquitin (conj. ubiquitin) from A. n = 4 for endogenous and WT TDP-43, n = 3 for M337V TDP-43, n = 3 for tau, n = 6 for α-syn, error bars represent SEM, * P < 0.05, ** P < 0.01, *** P < 0.001.

Previous studies [22, 27] have reported findings regarding effects of USP14 overexpression or inhibition on degradation of aggregation-prone proteins in neurodegenerative diseases. We repeated the experiments by co-expressing the empty vector control, USP14 WT or C114A with myc-TDP-43, tau or α-synuclein in HEK293T cells (Fig 1A and 1B). Neither USP14 WT nor C114A changed the protein level of endogenous TDP-43, myc-TDP-43 WT, myc-TDP-43 M337V (an ALS causal mutation, [32]), tau or α-synuclein.

We also tested two published USP14 inhibitors, IU1 [22] and b-AP15 [29] in the ubiquitin-rhodamine hydrolysis assay with purified USP14 in the presence of ubiquitin vinyl sulphone treated 26S proteasome [33] (S1 Fig). IU1 inhibited USP14 with an IC_50_ of 5.51 μM as previously published [22] whereas b-AP15 did not show significant inhibition up to 100 μM (S1 Fig). A related compound VLX1570 also did not inhibit USP14 reconstituted with Ub-Vs treated proteasome for unknown reasons [34]. IU1 treatment (75 μM, 6 hours) did not affect the level of TDP-43, tau or α-synuclein expressed in HEK293T cells (S2A and S2B Fig). Proteasome inhibitors (MG132 10 μM + PS341 10 μM, 6 hours) as expected caused a robust accumulation of ubiquitinated proteins but did not affect the level of TDP-43, tau or α-synuclein (S2A and S2B Fig). Dose responses of 1U1 up to 100 μM (6 hours) also did not affect the level of TDP-43, tau or α-synuclein (S2C and S2D Fig). b-AP15 treatment caused an accumulation of ubiquitinated proteins as previously published [29] but did not affect the level of TDP-43, tau or α-synuclein (S2E and S2F Fig).

Although neither USP14 C114A nor USP14 inhibitors affected the levels of TDP-43, tau or α-synuclein, USP14 C114A expression consistently caused robust accumulation of ubiquitinated proteins in HEK293T cells in all the experiments (Fig 1).

### Inactive USP14 C114A causes accumulation of ubiquitinated proteins in HEK293T cells

**Fig 2:**
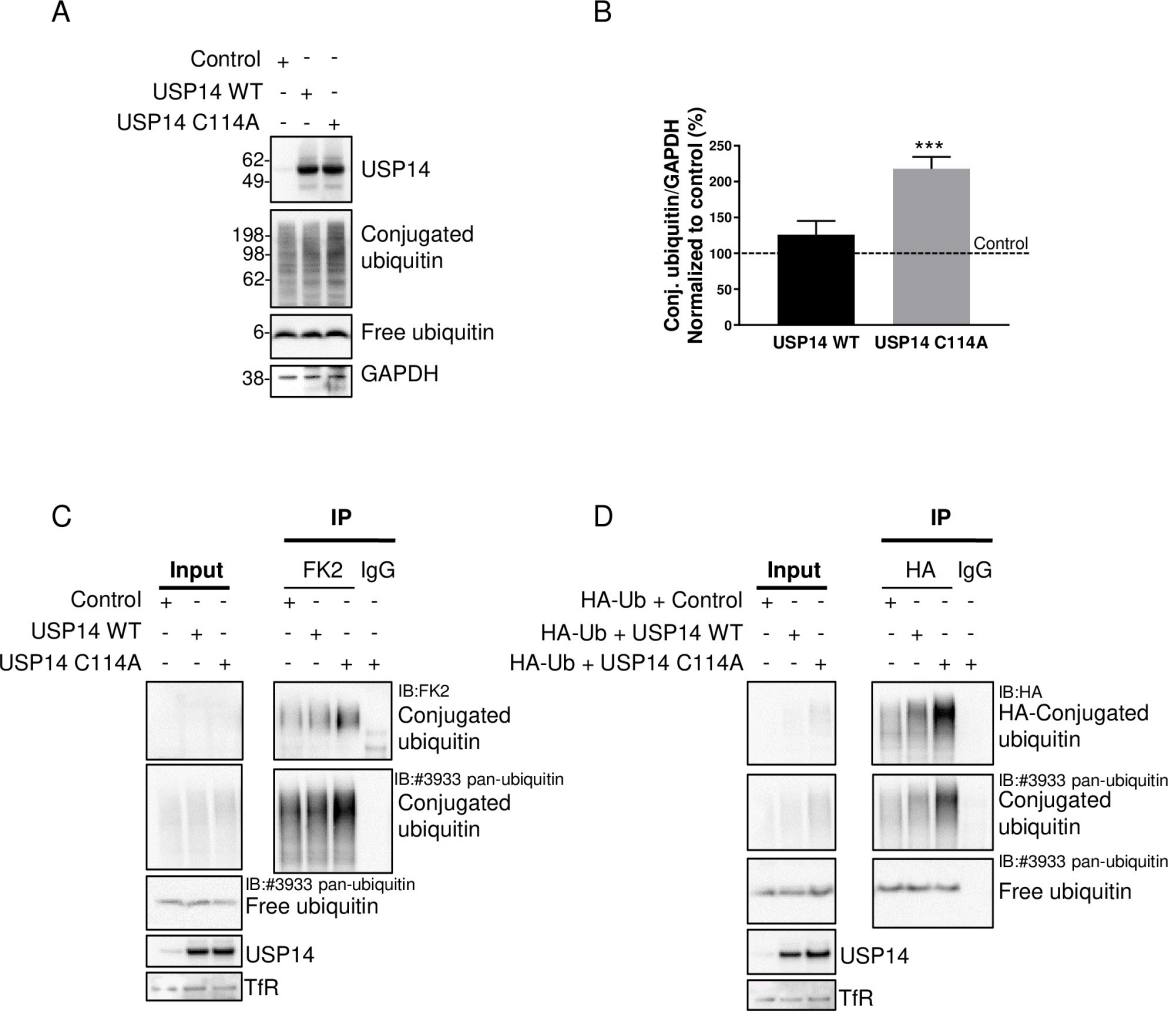
Inactive USP14 C114A causes accumulation of ubiquitinated proteins in HEK293T cells. (A) Immunoblot showing expression of USP14 C114A in HEK293T cells causes an accumulation of ubiquitinated proteins. (B) Quantification of conjugated ubiquitin from (A). *** *P* < 0.001, n = 4, error bars represent SEM. (C) Ubiquitin IP with the FK2 antibody from the cells expressing the indicated proteins, followed by IB with FK2 and the pan-ubiquitin antibody #3933. (D) IP using the HA antibody from the cells co-expressing HA-ubiquitin with the indicated proteins, followed by IB with the HA and pan-ubiquitin antibodies.

We next expressed the empty vector, USP14 WT or C114A in HEK293T cells to understand whether USP14 C114A has the same effect on ubiquitinated proteins in the absence of co-expressed TDP-43, tau or α-synuclein. USP14 C114A alone caused a similar accumulation of ubiquitinated proteins (Fig 2A and 2B). Using an immunoprecipitation compatible ubiquitin antibody FK2, we were able to pull down more ubiquitin conjugates from the cells expressing USP14 C114A compared to the empty vector control or WT (Fig 2C). Furthermore, using an HA antibody, we were able to pull down more ubiquitin conjugates from the cells co-transfected with HA-ubiquitin and USP14 C114A compared to the cells co-transfected with the empty vector control or WT (Fig 2D). These data confirm inactive USP14 causes ubiquitinated protein accumulation in cells.

### Identification of the accumulated ubiquitinated proteins induced by USP14 C114A

**Fig 3:**
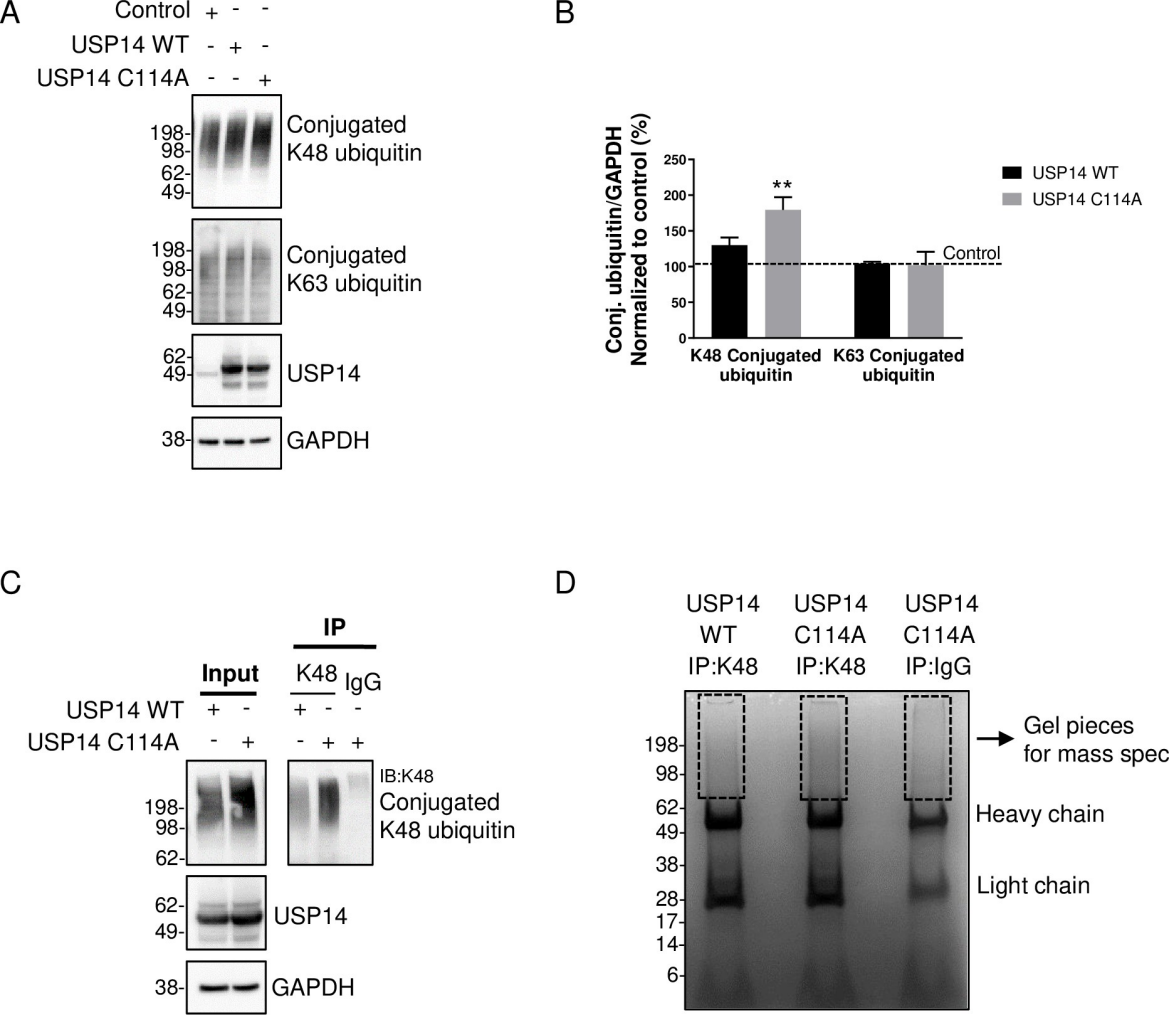
Isolation of the ubiquitinated proteins accumulated in the cells expressing USP14 C114A. (A) Immunoblot showing K48-linked but not K63-linked ubiquitinated proteins are accumulated in the cells expressing USP14 C114A. (B) Quantification of protein levels from (A). n = 3, ** *P* < 0.01, error bars represent SEM. (C) and (D) K48-linked ubiquitinated proteins were immunoprecipitated from the cells expressing the indicated proteins. A control IP was done with IgG from the cells expressing USP14 C114A. The IP samples were subjected to (C) IB with the K48 antibody or (D) Coomassie blue staining. The gel pieces above the antibody heavy chain in (D) were cut for mass spectrometry analysis.

We next assessed the chain linkage of the ubiquitin conjugates accumulated with USP14 C114A expression. We focused on lysine 48 (K48)- and lysine 63 (K63)-linked chains as these are the most abundant ubiquitin chain types in cells [35]. K48-linked but not K63-linked ubiquitin conjugates accumulated in the cells expressing USP14 C114A (Fig 3A and 3B). To identify these proteins, we combined immunoprecipitation with the antibody against K48-linked ubiquitin chains (simplified as K48 antibody hereafter) and mass spectrometry (IP-MS). K48-linked ubiquitinated proteins were immunoprecipitated from the cells expressing USP14 WT or C114A and separated by SDS-PAGE, followed by immunoblotting (IB) (Fig 3C) or staining with Coomassie blue (Fig 3D), both of which showed more ubiquitinated proteins were pulled down from the USP14 C114A than WT samples. We also included a control IgG IP from USP14 C114A expressing cells to be able to filter out proteins that were pulled down non-specifically (Fig 3C and 3D). The lanes were cut above the antibody heavy chain and subjected to MS analysis (Fig 3D). The IP-MS was repeated in triplicate and all the proteins identified in the IgG IP were removed without further consideration. The remaining proteins more abundant in the USP14 C114A than WT samples in all three replicates are listed in Table 1 (details of individual repeats in S1 Table).

Among the list are well-established proteasomal substrates and important signaling molecules such as β-catenin [36] and Argonaute-2 [37] (Table 1). Multiple proteasome subunits were identified including PSMA4 (20S subunit α3), PSMC1 (19S AAA-ATPase subunit Rpt2), PSMC3 (19S AAA-ATPase subunit Rpt5), and PSMD4 (19S non-ATPase subunit Rpn10/S5A). The Angelman syndrome disease gene ubiquitin-protein ligase E3A (UBE3A) [38] and mental retardation disease gene ubiquitin specific peptidase, X-linked (USP9X) [39] were also identified. The IP-MS data suggest USP14 regulates not only substrate deubiquitination but also the ubiquitination status of the proteasome.

### Confirmation that ubiquitinated β-catenin accumulates in USP14 C114A expressing cells

**Fig 4:**
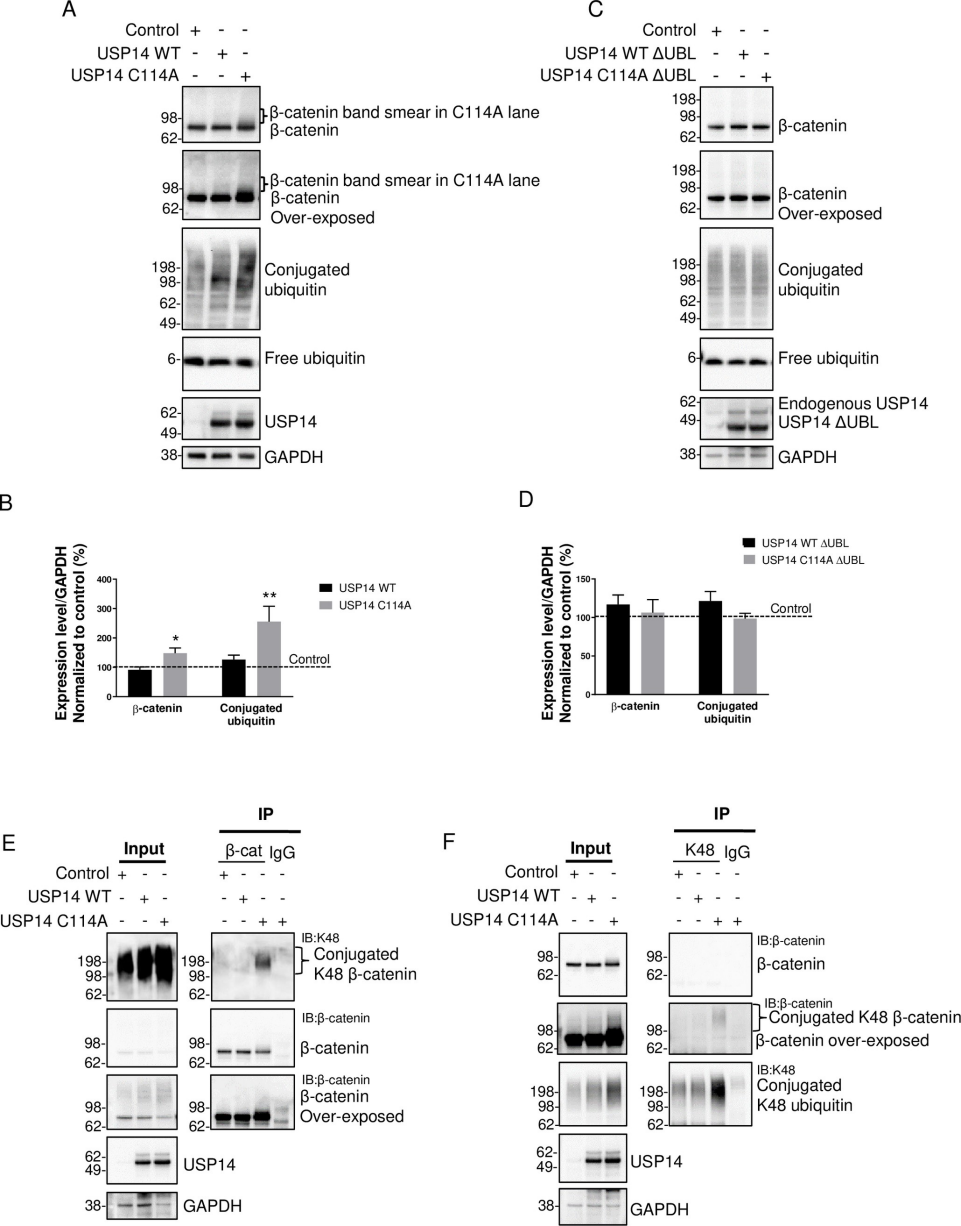
Confirmation that ubiquitinated β-catenin accumulates in USP14 C114A expressing cells. (A) Immunoblot showing USP14 C114A increases the total protein level of β-catenin in cells. Note the smearing of the β-catenin band in the C114A lane. (B) Quantification from (A). n = 6, * *P* < 0.05, ** *P* < 0.01, error bars represent SEM. (C) Immunoblot showing proteasome binding is required for the effects of USP14 C114A on β-catenin and conjugated ubiquitin. Deletion of the UBL proteasome binding domain (ΔUBL) abolishes the effects of USP14 C114A on β-catenin and conjugated ubiquitin. (D) Quantification from (C). n = 6, error bars represent SEM. (E) β-catenin IP followed by K48 IB showing ubiquitinated β-catenin is accumulated in the cells expressing USP14 C114A. (F) K48 IP followed by β-catenin IB showing ubiquitinated β-catenin is accumulated in the cells expressing USP14 C114A.

We focused on β-catenin to validate the IP-MS data. A higher molecular weight smear of the β-catenin band was observed in the USP14 C114A samples, suggesting an accumulation of ubiquitinated β-catenin (Fig 4A). The total protein level of β-catenin was increased in the cells expressing USP14 C114A compared to the empty vector control or WT (Fig 4A and 4B). The level of ubiquitin conjugates was also significantly increased as seen previously (Figs 2, 4A and 4B).

To understand if the effects of USP14 C114A are dependent on proteasome binding, we generated USP14 WT and C114A constructs lacking the UBL domain, USP14 WT ΔUBL and C114A ΔUBL. The ΔUBL constructs were predicted to be ~10 kDa smaller than the full-length constructs and were expressed as expected (Fig 4C). USP14 C114A ΔUBL had no effect on the levels of β-catenin or ubiquitin conjugates, suggesting that proteasome binding is required for USP14 C114A’s effect on ubiquitinated proteins (Fig 4C and 4D).

To further confirm the accumulation of ubiquitinated β-catenin in the cells expressing USP14 C114A, we conducted mutual IP-IB experiments using β-catenin or K48 antibodies (Fig 4E and 4F). β-catenin IP showed a smear of K48-linked ubiquitinated β-catenin from USP14 C114A expressing cells (Fig 4E). The reverse approach of K48 IP and β-catenin IB also showed a high molecular weight smear of ubiquitinated β-catenin from USP14 C114A expressing cells (Fig 4F). Taken together, these data confirm that ubiquitinated β-catenin accumulated in USP14 expressing cells.

### Inactive UCHL5 C88A causes accumulation of ubiquitinated proteins but does not affect β-catenin

**Fig 5:**
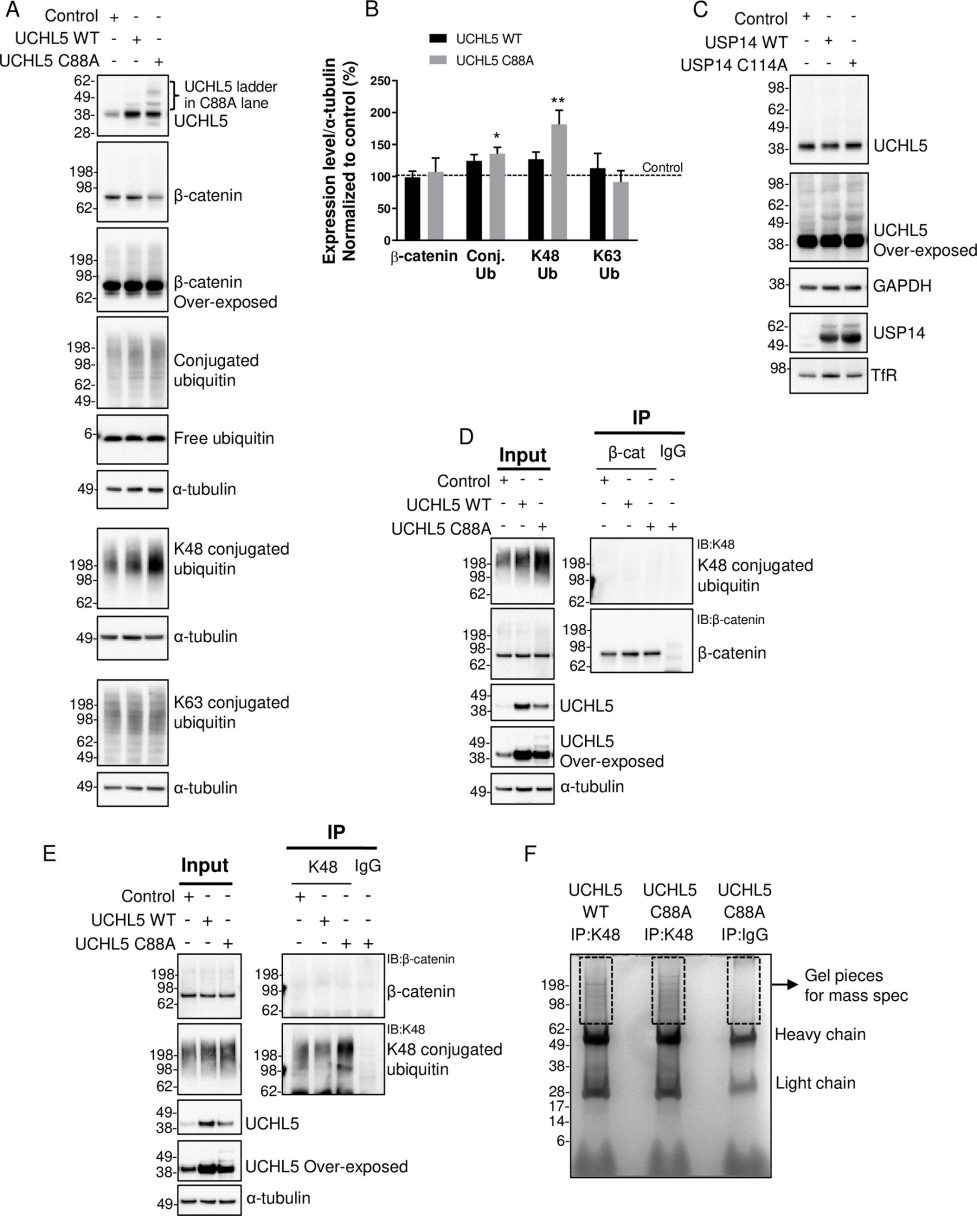
UCHL5 C88A causes an accumulation of ubiquitinated proteins but does not affect β-catenin degradation. (A) Immunoblot showing the effects UCHL5 WT and C88A in HEK293T cells. Note the lack of band smearing of β-catenin but the presence of band laddering of UCHL5 and the increase of conjugated ubiquitin in the C88A lane. (B) Quantification from (A). n = 3–6, * *P* < 0.05, ** *P* < 0.01, error bars represent SEM. (C) Immunoblot showing USP14 C114A does not induce UCHL5 band laddering in cells. (D) β-catenin IP followed by K48 IB showing the lack of ubiquitinated β-catenin accumulation in the cells expressing UCHL5 C88A. (E) K48 IP followed by K48 and β-catenin IB showing although the accumulated ubiquitinated proteins were immunoprecipitated but ubiquitinated β-catenin was not accumulated in the cells expressing UCHL5 C88A. (F) K48-linked ubiquitinated proteins were immunoprecipitated from the cells expressing the indicated proteins. A control IP was done with IgG from the cells expressing UCHL5 C88A. The IP samples were subjected to Coomassie blue staining. The gel pieces above the antibody heavy chain were cut for mass spectrometry analysis.

To understand whether USP14 has a specific effect on β-catenin or the other proteasomal cysteine-protease deubiquitinase UCHL5 also shares the same effect, we generated a catalytically inactive mutant of UCHL5 by mutating the catalytic cysteine at position 88 to alanine (UCHL5 C88A). This mutant was shown to lack deubiquitinase activity but bind to the proteasome similarly to WT [16]. Interestingly, expression of the inactive UCHL5 C88A also led to an accumulation of K48-linked ubiquitin conjugates (Fig 5A and 5B). Furthermore UCHL5 C88A expression led to a high molecular weight laddering of UCHL5 itself as detected by IB with a UCHL5 antibody, presumably representing ubiquitinated UCHL5 (Fig 5A) [40–42]. While the IP-MS data suggest USP14 is required for deubiquitination of multiple proteasomal subunits (Table 1), USP14 C114A did not affect UCHL5 ubiquitination as shown by the lack of the UCHL5 laddering (Fig 5C).

While UCHL5 C88A showed clear effects on ubiquitin conjugates and its own ubiquitination, it had no effect on β-catenin accumulation or band smearing (Fig 5A and 5B). To rule out the possibility that the accumulation of ubiquitinated β-catenin was below the detection limit of straight IB, we conducted mutual IP-IB experiments using β-catenin and K48 antibodies, similar to those done with USP14 (Figs 4E, 4F, 5D and 5E). β-catenin IP failed to pull down any K48-linked ubiquitinated β-catenin from UCHL5 C88A expressing cells although the input samples confirmed the accumulation of K48-linked ubiquitin conjugates and UCHL5 laddering in those cells (Fig 5D). K48 IP pulled down the accumulated ubiquitinated proteins from UCHL5 C88A expressing cells but β-catenin was not detected (Fig 5E). These data indicate inactive USP14 but not inactive UCHL5 specifically affects β-catenin ubiquitination, suggesting β-catenin could be a specific substrate of USP14.

### Identification of the accumulated ubiquitinated proteins induced by UCHL5 C88A

To identify the accumulated ubiquitinated proteins induced by UCHL5 C88A, K48-linked ubiquitinated proteins were immunoprecipitated from UCHL5 WT or C88A expressing cells and subjected to SDS-PAGE followed by Coomassie blue staining for MS (Fig 5F). Similarly to USP14, we included a control IgG IP from UCHL5 C88A expressing cells to filter out non-specific proteins. The IP-MS was repeated in triplicate and all the proteins identified in the IgG IP were removed without further consideration. The remaining proteins more abundant in UCHL5 C88A than WT samples in all three replicates are listed in Table 2 (details of individual repeats in S3 Table).

16 unique proteins were identified for USP14 and 23 for UCHL5, and 6 common proteins for both (Tables 1 and 2, Fig 7A). Importantly, ubiquitinated β-catenin was not enriched in UCHL5 C88A samples from mass spectrometry, which is consistent with the immunoblotting data (Figs 4 and 5). Moreover, ubiquitinated UCHL5 was indeed enriched in UCHL5 C88A samples but not in USP14 C114A samples from mass spectrometry, which is consistent with that the high molecular weight laddering of UCHL5 represented ubiquitinated species and suggests UCHL5 auto-deubiquitinates itself (Fig 5A). Previously known proteasome substrates which are also disease genes were identified for UCHL5 such as Ataxin-10 [43] and calreticulin [44]. Ubiquitinated proteasome subunits PSMA4 and PSMC3 (Rpt5) accumulated in both USP14 C114A and UCHL5 C88A samples, while ubiquitinated PSMC1 (Rpt2) and PSMD4 (Rpn10/S5A) were specific for USP14 and PSMD1 (Rpn2) was specific for UCHL5 (Fig 7A).

### Confirmation that ubiquitinated PSMC1 and PSMD4 accumulate in USP14 C114A expressing cells

**Fig 6:**
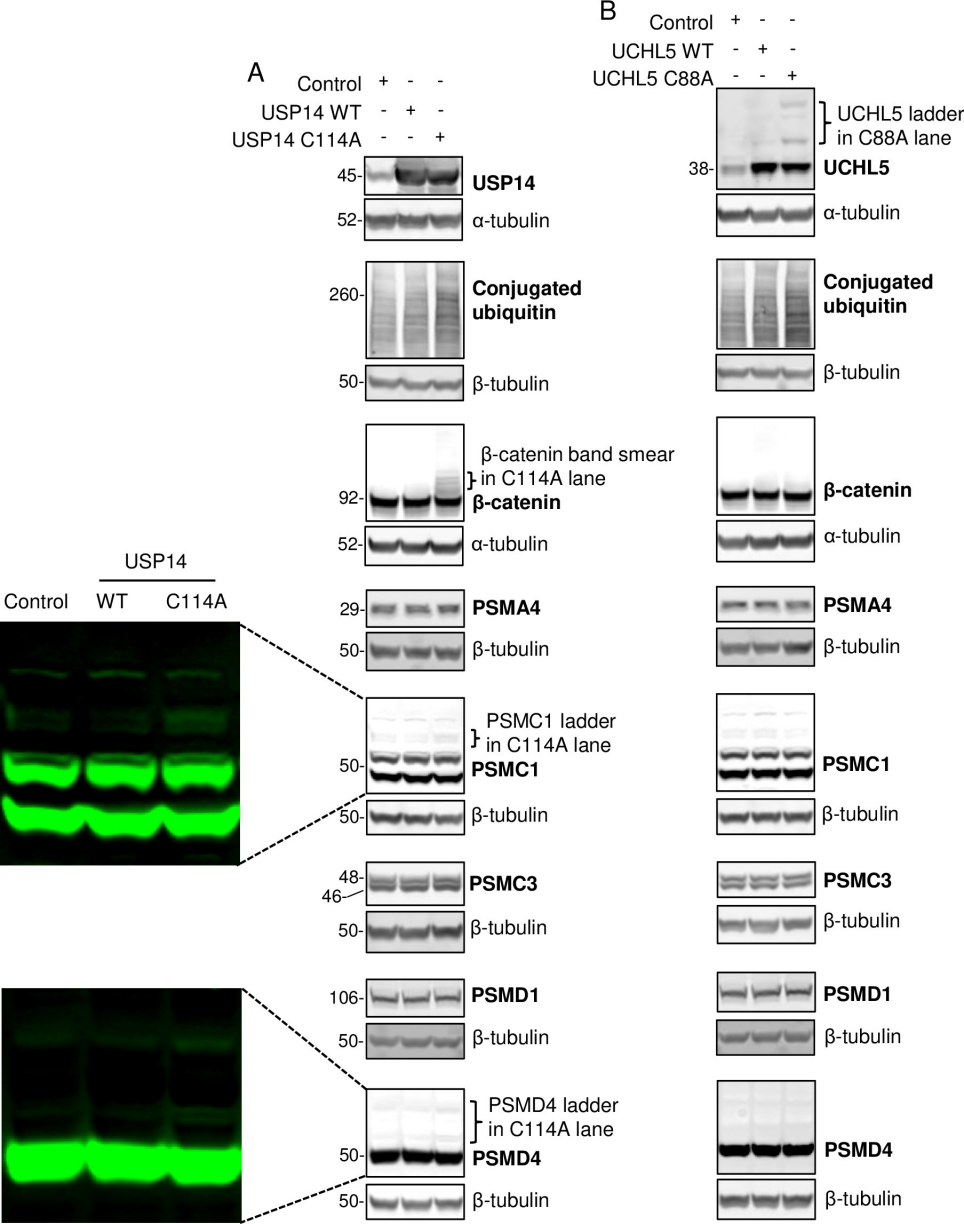
Confirmation that ubiquitinated PSMC1 and PSMD4 accumulate in USP14 C114A expressing cells. (A) Immunoblot showing effects of USP14 WT and C114A in HEK293T cells. Note the increased ubiquitin conjugates, the band smearing of β-catenin, and the band laddering of PSMC1 and PSMD4 in C114A samples. (B) Immunoblot showing effects of UCHL5 WT and C88A in HEK293T cells. Note the increased ubiquitin conjugates and the band laddering of UCHL5 in C88A samples.

**Fig 7:**
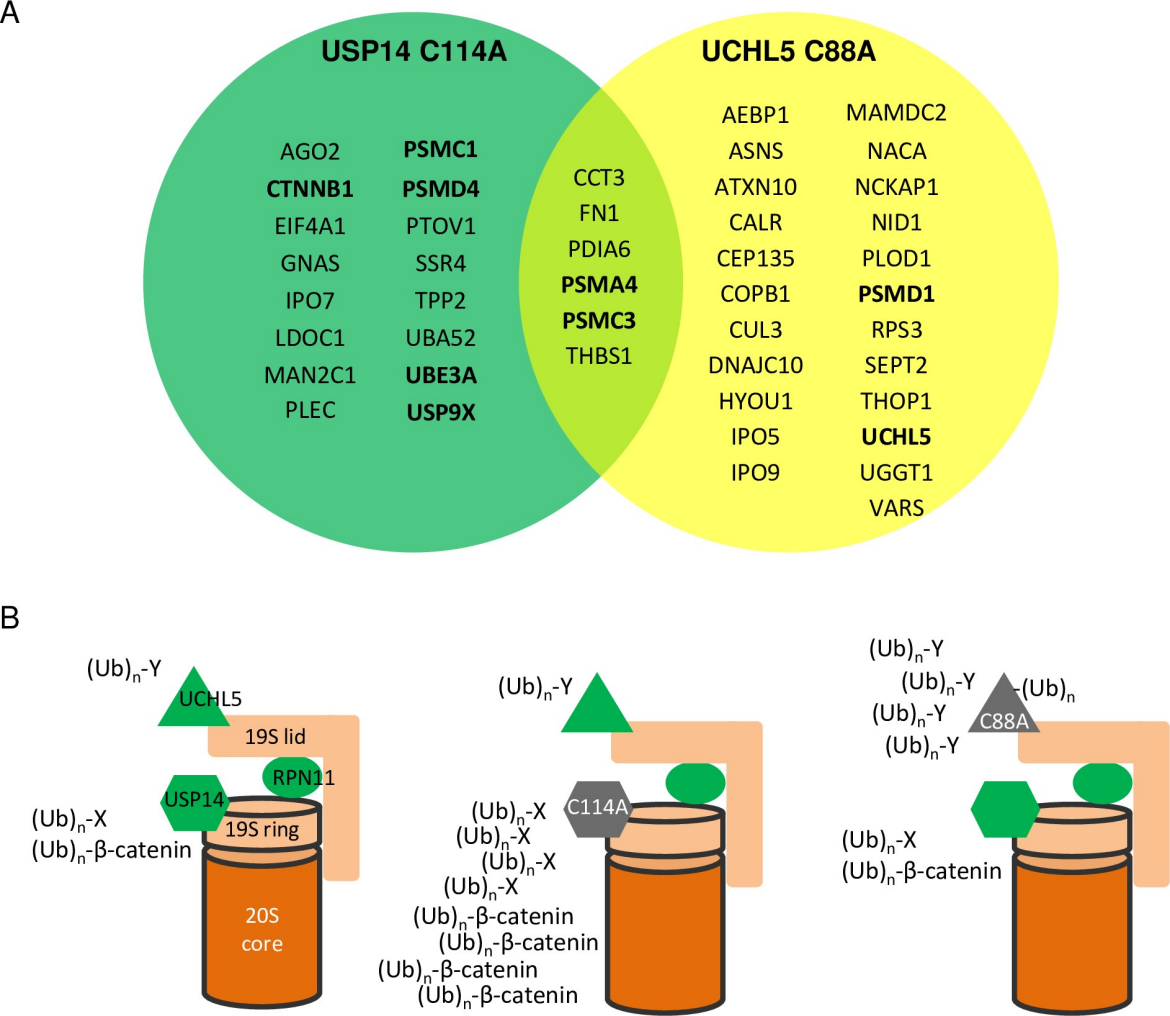
A model showing the effects of inactive USP14 and inactive UCHL5 at the proteasome. (A) Venn diagram of the ubiquitinated proteins accumulated in USP14 C114A and UCHL5 C88A expressing cells. (B) Inactive USP14 C114A causes accumulation of specific substrates (Ub)_n_-X including ubiquitinated β-catenin. Inactive UCHL5 C88A causes accumulation of specific substrates (Ub)_n_-Y and increases its own ubiquitination.

We lastly focused to verify that ubiquitinated proteasomal subunits accumulate in cells expressing USP14 C114A or UCHL C88A. Immunoblotting was performed with the antibodies against the proteasomal subunits identified by mass spectrometry (Tables 1 and 2, Fig 7A). The detection of the ubiquitinated ladder is dependent on a high affinity antibody and on that ubiquitination does not interfere with antibody binding in immunoblotting experiments. Ladders of PSMC1 and PSMD4, presumably representing ubiquitinated species, were observed in USP14 C114A but not UCHL5 C88A samples (Fig 6). This data is consistent with the mass spectrometry identification (Tables 1 and 2, Fig 7A). The immunoblotting signals were weak but consistent. The original fluorescent images were included for better visualization (Fig 6A). The antibodies against PSMA4, PSMC3, and PSMD1 only detected the major protein bands even with long exposure (Fig 6), therefore different antibodies need to be tested in the future. The robust accumulation of ubiquitin conjugates, ubiquitinated β-catenin, and ubiquitinated UCHL5 were clearly observed in this set of experiments as seen previously (Figs 2–6).

## Discussion

In the absence of a ubiquitinated substrate, USP14/Ubp6 reduces basal peptidase and ATPase activity of the proteasome to degrade unstructured proteins lacking ubiquitination [20], supporting its function as a check point to prevent non-specific degradation and to allow entry of proper substrates. Ubiquitin loaded USP14 activates proteasomal ATPases, causes 20S gate opening, but inhibits RPN11, and delayed degradation of ubiquitinated substrates [20, 21, 45–47], supporting that it ensures sequential deubiquitination by itself first and then RPN11, and coordinates substrate unfolding and entry into the 20S CP. When loaded with a ubiquitinated substrate, UCHL5 also activates ATPase activity and 20S gate opening of the proteasome purified from USP14 knockout mammalian cells [46], suggesting UCHL5 may function as another check point to coordinate substrate entry and processing. USP14 and UCHL5 associate with the proteasome through different ubiquitin receptors, USP14 to Rpn1 at the base of the 19S RP and UCHL5 to Rpn13/ADRM1 at the top of the 19S RP [1] (Fig 7B). Both USP14 and UCHL5 play complex catalytic and allosteric roles during substrate degradation by the proteasome but it is unclear whether they process different substrates at the proteasome.

In this study we identified that the inactive mutants USP14 C114A and UCHL5 C88A caused accumulations of distinct sets of ubiquitinated proteins in HEK293T cells (Figs 2–6, Tables 1 and 2). Both mutants retain the activity to bind to the proteasome [16, 48]. The UBL proteasome-binding domain of USP14 is required for C114A’s effect on ubiquitinated proteins (Fig 4C). We confirmed the specific accumulation of ubiquitinated β-catenin, PSMC1 and PSMD4 in USP14 C114A expressing cells (Figs 4 and 6, Table 1), and that UCHL5 activity is required for its auto-deubiquitination (Figs 5 and 6, Table 2).

In our mass spectrometry experiments, although the ubiquitinated proteins were enriched by ubiquitin immunoprecipitation, their abundance in general was low. Therefore we faced the issues of low number of peptides identified for each protein and signal-to-noise being close to the detection limit, and the resulted variability. We mitigated these issues by independent replications and by inclusion of IgG control as discussed below, but orthogonal experiments will be needed to validate the hits identified in Tables 1 and 2 for USP14 and UCHL5.

For practical reasons such as limited availability of suitable antibodies, it is difficult to verify all the proteins that were identified by mass spectrometry with immunoblotting or immunoprecipitation experiments, but a few pieces of evidence strongly supports the validity of the data sets. The IP-MS experiments for both USP14 (Fig 3D) and UCHL5 (Fig 5F) were repeated three times. For each experiment, the IgG control IP helped firstly remove the proteins non-specifically pulled down. Then the proteins more abundant in the mutant than the WT samples were identified for each repeat. Only the common hits in all three replicates were included in Tables 1 and 2 (see S1 and S3 Tables for individual repeats). This process should remove significant amount of random noise from the mass spectrometry data. As control, we also performed inverted analyses to identify proteins less abundant in the mutant than the WT samples in all three replicates (S2 and S4 Tables). 5 proteins were less abundant versus 22 more abundant in USP14 C114A than WT samples. 1 protein was less abundant versus 29 more abundant in UCHL5 C88A than WT sample. Furthermore, the mass spectrometry data correlate well with individual protein verification. Ubiquitinated β-catenin, PSMC1, and PSMD4 were identified to be more abundant by mass spectrometry in USP14 C114A but not UCHL5 C88A samples (Tables 1 and 2, Fig 7A). Their ubiquitinated smear or ladders were only detected in USP14 C114A but not UCHL5 C88A cell lysates (Figs 4 and 6). While ubiquitinated UCHL5 was identified to be more abundant by mass spectrometry in UCHL5 C88A but not USP14 C114A samples (Fig 7A). Its ubiquitinated ladder was only detected in UCHL5 C88A but not USP14 C114A cell lysates (Fig 5). While both USP14 C114A and UCHL5 C88A induced robust accumulation of ubiquitinated proteins, our data strongly support they each can affect distinct protein substrates.

USP14 dynamically associates with the proteasome [14]. Higher than endogenous expression levels (Fig 2A) are expected to increase the percentage of USP14-containing proteasomes in the cells over-expressing USP14 WT or C114A [49, 50]. It was reported that the active site cysteine to alanine mutations increase the binding affinity of ubiquitin to a few DUBs [51]. Non-proteasomal DUBs when mutated at the active site cysteine (to alanine) may sequester ubiquitinated proteins from degradation. However as USP14 is a proteasomal DUB, ubiquitinated substrates may be stuck at the proteasome containing USP14 C114A (Fig 7B). This hypothesis is consistent with the observation that increased ubiquitinated proteins are associated with the proteasome pulled down from the cells expressing USP14 C114A [51]. UCHL5 is associated with both the proteasome and the INO80 chromatin remodeling complex, and functions in both contexts [52]. Specific complex pulldown experiments need to be done to understand where in the cell UCHL5 C88A causes ubiquitinated protein accumulation.

Besides known proteasomal substrates, 5 ubiquitinated proteasomal subunits, PSMA4 (20S subunit α3), PSMC1 (Rpt2), PSMC3 (Rpt5), PSMD1 (Rpn2) and PSMD4 (Rpn10/S5A), were identified to be enriched in USP14 C114A or UCHL5 C88A samples (Figs 6 and 7A). It was reported that ubiquitination of Rpt5, Rpn10/S5A, Rpn13/ADRM1, and UCHL5 is induced by proteasome-associated UBE3A and UBE3C at the purified proteasome [41]. Ubiquitin aldehyde further increases ubiquitination of these proteins, demonstrating USP14 and UCHL5 antagonize UBE3A and UBE3C to deubiquitinate these proteins at the proteasome [41]. In our study, ubiquitinated PSMC1 and PSMD4 specifically accumulated in USP14 C114A samples, while ubiquitinated PSMD1 specifically accumulated in UCHL5 C88A samples (Fig 7A). Interestingly, PSMC1 (Rpt2) is in close proximity to the ubiquitin receptor Rpn1 which recruits USP14 to the proteasome, and PSMD1 (Rpn2) is in close proximity to the ubiquitin receptor Rpn13 which recruits UCHL5 to the proteasome [53]. This may provide a structural explanation to the proteasomal subunit specificity we observed between USP14 and UCHL5. Ubiquitination of proteasome subunits impairs substrate recognition and processing by the proteasome and cellular stresses can modulate the ubiquitination status of the proteasome [41, 42]. Together our data suggest that USP14 and UCHL5 may regulate the proteasome activity by specifically modulating the ubiquitination status of the proteasome subunits. The *in vitro* enzyme assays with purified proteasome [41] suggests that the proteasome subunits deubiquitination by USP14 and UCHL5 occurs at the proteasome, but future experiments such as isolating the proteasome from cells expressing USP14 or UCHL5 are required to clarify whether the deubiquitination occurs at the proteasome or on free proteasomal subunits.

Ubiquitinated UCHL5 was found to be enriched in UCHL5 C88A samples by mass spectrometry analysis (Table 2, S3 Table). Consistently, the high molecular weight ladder of UCHL5 C88A was detected in our study (Figs 5A and 6B) and by others ([40]. A DUB closely related to UCHL5, BAP1 can also auto-deubiquitinate to regulate its own cellular localization and function [54]. A structural study shows that UCHL5 can form a tetramer, or a dimer of dimers [55], suggesting the auto-deubiquitination could be intramolecular or intermolecular. Interesting questions, such as how ubiquitination affects UCHL5’s activity and recruitment to the proteasome and INO80 complex, await future studies.

A recent study, combining USP14 interactors, up-regulated ubiquitination sites and down-regulated proteins in response to USP14 knockdown, identified fatty acid synthase (FASN) as a specific USP14 substrate and suggested a list of proteins as potential USP14 substrates [56]. In our study, FASN was pulled down non-specifically by the IgG control in all repeats so it was not proceeded further for the comparison between WT and mutant samples (data not shown). However, a few proteasomal subunits were also identified in that study [56], including PSMC1 and PSMC3 which were identified in our study for USP14 as well (Table 1). It is important to distinguish the roles of USP14 in degradation inhibition by removing the ubiquitin chains of substrates, in degradation facilitation by deubiquitinating substrates to allow unfolding, and in regulation of the proteasomal ubiquitination in situ.

We were interested in the effect of USP14 inhibition on reducing neurodegenerative disease proteins such as α-synuclein, TDP-43, and tau, but neither USP14 inhibitors nor USP14 C114A showed consistent and significant effects on these proteins in HEK293T cells (Fig 1 and S2 Fig). Similar findings were also reported by Miller and colleagues [27]. TDP-43 and α-synuclein have long half-lives and it is demonstrated that autophagy plays an important role in their degradation, and both proteasome and autophagy appear to be important for tau degradation [57–59]. Consistently, treating cells with proteasome inhibitors did not modulate the levels of TDP-43, tau or α-synuclein (S2 Fig). These data raise the question whether a proteasome related approach can efficiently modulate the level of these aggregation-prone proteins.

In a phenotypic screen for compounds that induce the lysosomal apoptosis pathway, b-AP15 was identified as a proteasomal inhibitor [29]. *In vitro* activity probe assays and cellular thermal shift assays show b-AP15 and related compounds inhibit both USP14 and UCHL5 [60, 61]. A related compound VLX1570 inhibited another DUB USP5 but did not inhibit USP14 or UCHL5 reconstituted with Ub-Vs treated proteasome [34]. This is consistent with our data that b-AP15 did not inhibit USP14 reconstituted with Ub-Vs treated proteasome (S1 Fig). In our study, the proteasome was isolated from mammalian cells and treated with Ub-Vs to remove the associated cysteine protease DUB activities. Then purified USP14 was added to the treated proteasome to ensure detected DUB activity was solely from USP14. This is a well established method to assess USP14 or UCHL5 activity with the proteasome *in vitro* [34]. With these data, the exact mechanism of action and specificity of b-AP15 remain unclear. IU1 shows a clear selectivity on USP14 over other DUBs, but a more broader off-target profile is unknown [34]. Unlike many kinase inhibitors that inhibit substrate phosphorylation in cells, IU1 has not been shown to increase ubiquitination of a substrate in cells. Therefore its actual cellular potency is unknown. At higher concentrations, IU1 showed cytotoxicity (data not shown) and neurotoxicity [28]. In our study, we did not observe a clear effect of IU1 or b-AP15 on accelerating the degradation of TDP-43, tau or α-synuclein (S2 Fig). Also IU1 (75 μM, 24 hours of treatment) did not affect β-catenin or conjugated ubiquitin in HEK293T cells (data not shown). IU1 and b-AP15 may have cell type specific effects, but cautions need to be taken when using these compounds to study USP14 or UCHL5 biology in cells due to their off-target effects, complex inhibition mechanisms and lack of immediate biomarkers such as substrate ubiquitination.

## Supporting information

S1 Fig The activity of 1U1 and b-AP15 on USP14 in the ubiquitin-rhodamine hydrolysis assay. The activity of 1U1 and b-AP15 on USP14 were examined *in vitro* in the ubiquitin-rhodamine hydrolysis assay in the presence of proteasome. The IC_50_ of IU1 is consistent as previously reported. The activity of b-AP15 was not detected. Calculated IC_50_ values were based on the average of three replicates. RFU, relative fluorescence units.
(TIF)

S2 Fig USP14 inhibition using the small molecule inhibitors IU1 or b-AP15 does not change the levels of TDP-43, tau or α-synuclein in HEK293T cells. (A) Immunoblot showing the effects of 75 μM IU1 treatment in the presence or absence of proteasome inhibitors (MG132 10 μM + PS341 10 μM) for 6 hours in HEK293T cells expressing myc-TDP-43, tau or α-synuclein. Ptsm inhibitors, proteasome inhibitors.
(B) Quantification of protein levels from A. n = 3, error bars represent SEM. 1U1 does not change the levels of TDP-43, tau or α-synuclein.
(C) Immunoblot showing the effects of 25, 50, 75 or 100 μM of IU1 treatment for 6 hours in HEK293T cells expressing myc-TDP-43, tau or α-synuclein.
(D) Quantification of protein levels from C. n = 3 for TDP-43 and tau, error bars represent SEM. n = 2 for α-synuclein, error bars represent range. No concentration response of 1U1 was observed on the levels of TDP-43, tau or α-synuclein.
(E) Immunoblot showing the effects of 0.5, 1 or 2 μM of b-AP15 treatment for 4 hours in HEK293T cells expressing myc-TDP-43, tau or α-synuclein.
(F) Quantification of protein levels from E. n = 3, * *P* < 0.05, *** *P* < 0.001, error bars represent SEM. b-AP15 causes accumulation of polyubiquitinated proteins and polyubiquitinated TDP-43. b-AP15 does not reduce the levels of TDP-43, tau or α-synuclein.
(TIF)

S1 Table Quantification of polyubiquitinated proteins more abundant in the USP14 C114A than WT samples in all three replicates. (XLSX)

S2 Table Quantification of polyubiquitinated proteins less abundant in the USP14 C114A than WT samples in all three replicates. (XLSX)

S3 Table Quantification of polyubiquitinated proteins more abundant in the UCHL5 C88A than WT samples in all three replicates. (XLSX)

S4 Table Quantification of polyubiquitinated proteins less abundant in the UCHL5 C88A than WT samples in all three replicates. (XLSX)
