# Supplementary figures and images for: Inactive USP14 and inactive UCHL5 cause accumulation of distinct ubiquitinated proteins in mammalian cells
Source: PLoS One. 2019 Nov 8;14(11):e0225145. doi: 10.1371/journal.pone.0225145 (PMC6839854; doi:10.1371/journal.pone.0225145)

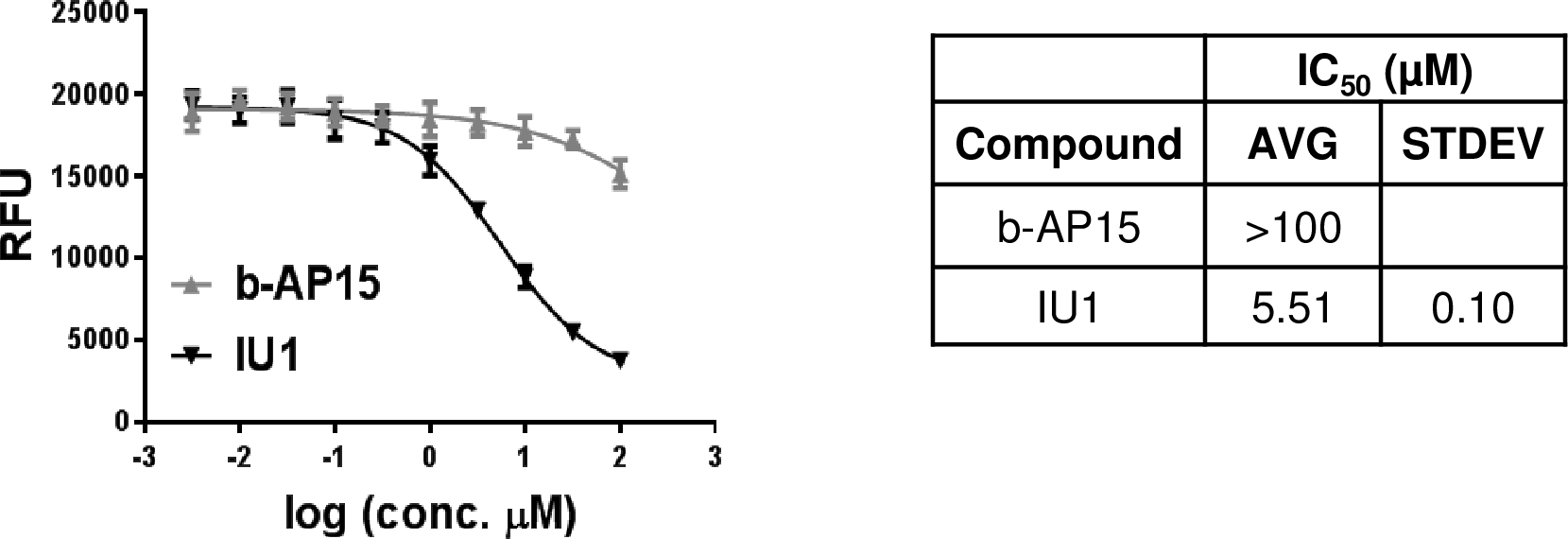

Supplement: S1 Fig — The activity of 1U1 and b-AP15 on USP14 were examined in vitro in the ubiquitin-rhodamine hydrolysis assay in the presence of proteasome. The IC50 of IU1 is consistent as previously reported. The activity of b-AP15 was not detected. Calculated IC50 values were based on the average of three replicates. RFU, relative fluorescence units. (TIF) [file pone.0225145.s001.tif]

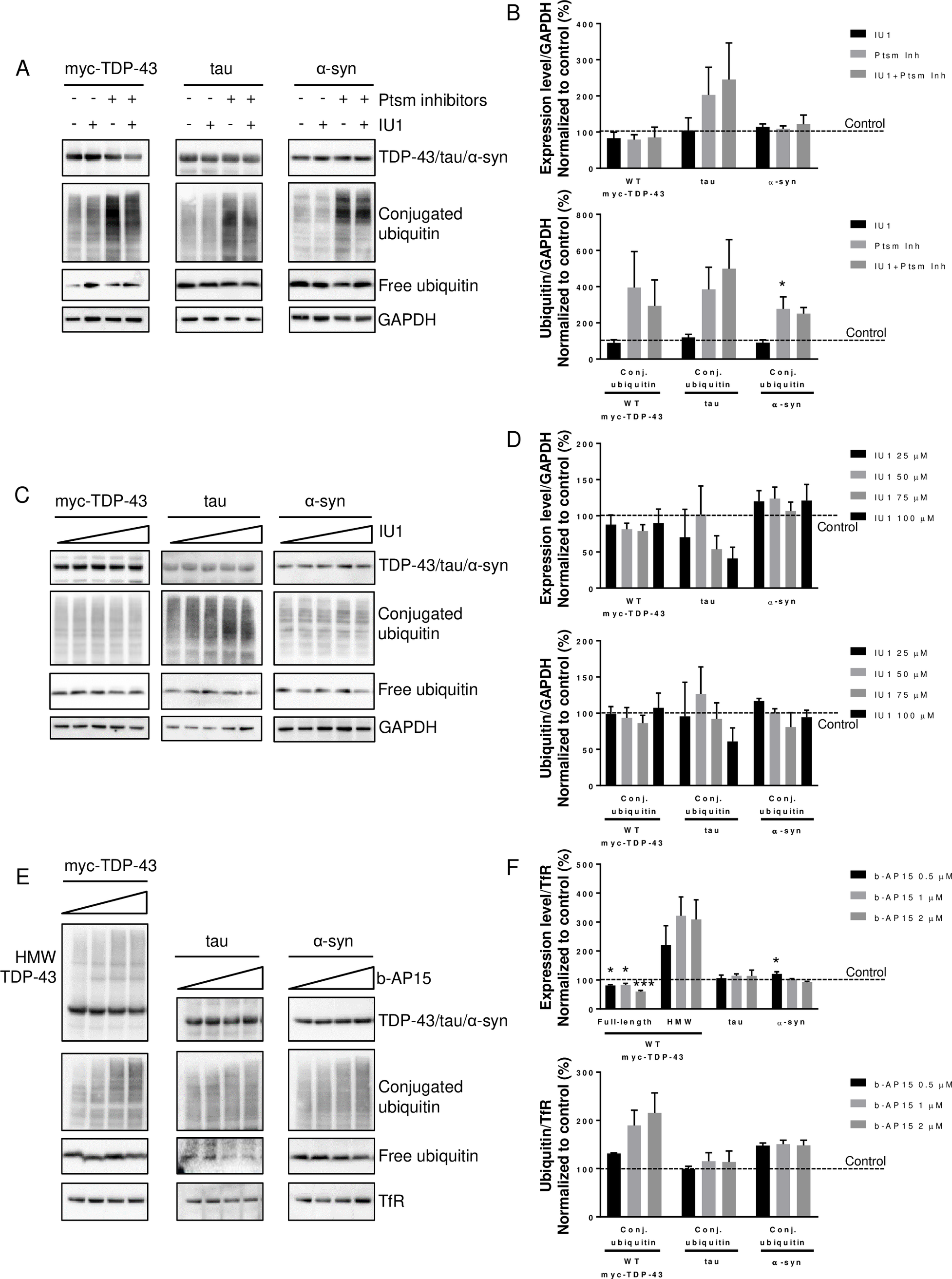

Supplement: S2 Fig — (A) Immunoblot showing the effects of 75 μM IU1 treatment in the presence or absence of proteasome inhibitors (MG132 10 μM + PS341 10 μM) for 6 hours in HEK293T cells expressing myc-TDP-43, tau or α-synuclein. Ptsm inhibitors, proteasome inhibitors. (B) Quantification of protein levels from A. n = 3, error bars represent SEM. 1U1 does not change the levels of TDP-43, tau or α-synuclein. (C) Immunoblot showing the effects of 25, 50, 75 or 100 μM of IU1 treatment for 6 hours in HEK293T cells expressing myc-TDP-43, tau or α-synuclein. (D) Quantification of protein levels from C. n = 3 for TDP-43 and tau, error bars represent SEM. n = 2 for α-synuclein, error bars represent range. No concentration response of 1U1 was observed on the levels of TDP-43, tau or α-synuclein. (E) Immunoblot showing the effects of 0.5, 1 or 2 μM of b-AP15 treatment for 4 hours in HEK293T cells expressing myc-TDP-43, tau or α-synuclein. (F) Quantification of protein levels from E. n = 3, * P < 0.05, *** P < 0.001, error bars represent SEM. b-AP15 causes accumulation of polyubiquitinated proteins and polyubiquitinated TDP-43. b-AP15 does not reduce the levels of TDP-43, tau or α-synuclein. (TIF) [file pone.0225145.s002.tif]
